# Patient-Safety-Related Hospital Deaths in England: Thematic Analysis of Incidents Reported to a National Database, 2010–2012

**DOI:** 10.1371/journal.pmed.1001667

**Published:** 2014-06-24

**Authors:** Liam J. Donaldson, Sukhmeet S. Panesar, Ara Darzi

**Affiliations:** 1 Institute of Global Health Innovation, Imperial College London, London, United Kingdom; 2 Department of Primary Care and Public Health, Imperial College London, London, United Kingdom; 3 Department of Surgery and Cancer, Imperial College London, London, United Kingdom; Brigham and Women′s Hospital, United States of America

## Abstract

**Background:**

Hospital mortality is increasingly being regarded as a key indicator of patient safety, yet methodologies for assessing mortality are frequently contested and seldom point directly to areas of risk and solutions. The aim of our study was to classify reports of deaths due to unsafe care into broad areas of systemic failure capable of being addressed by stronger policies, procedures, and practices. The deaths were reported to a patient safety incident reporting system after mandatory reporting of such incidents was introduced.

**Methods and Findings:**

The UK National Health Service database was searched for incidents resulting in a reported death of an adult over the period of the study. The study population comprised 2,010 incidents involving patients aged 16 y and over in acute hospital settings. Each incident report was reviewed by two of the authors, and, by scrutinising the structured information together with the free text, a main reason for the harm was identified and recorded as one of 18 incident types. These incident types were then aggregated into six areas of apparent systemic failure: mismanagement of deterioration (35%), failure of prevention (26%), deficient checking and oversight (11%), dysfunctional patient flow (10%), equipment-related errors (6%), and other (12%). The most common incident types were failure to act on or recognise deterioration (23%), inpatient falls (10%), healthcare-associated infections (10%), unexpected per-operative death (6%), and poor or inadequate handover (5%). Analysis of these 2,010 fatal incidents reveals patterns of issues that point to actionable areas for improvement.

**Conclusions:**

Our approach demonstrates the potential utility of patient safety incident reports in identifying areas of service failure and highlights opportunities for corrective action to save lives.

*Please see later in the article for the Editors' Summary*

## Abstract 2

Sukhmeet Panesar and colleagues classified reports of patient-safety-related hospital deaths in England to identify patterns of cases where improvements might be possible.

*Please see later in the article for the Editors' Summary*

## Editors' Summary

**Background:**

Being admitted to the hospital is worrying for patients and for their relatives. Will the patient recover or die in the hospital? Some seriously ill patients will inevitably die, but in an ideal world, no one should die in the hospital because of inadequate or unsafe care (an avoidable death). No one should die, for example, because healthcare professionals fail to act on signs that indicate a decline in a patient's clinical condition. Hospital mortality (death) is often regarded as a key indicator of patient safety in hospitals, and death rate indicators such as the “hospital standardized mortality ratio” (the ratio of the actual number of acute in-hospital deaths to the expected number of in-hospital deaths) are widely used to monitor and improve hospital safety standards. In England, for example, a 2012 report that included this measure as an indicator of hospital performance led to headlines of “worryingly high” hospital death rates and to a review of the quality of care in the hospitals with the highest death rates.

**Why Was This Study Done?:**

Hospital standardized mortality ratios and other measures of in-patient mortality can be misleading because they can, for example, reflect the burden of disease near the hospital rather than the hospital's quality of care or safety levels. Moreover, comparative data on hospital mortality rates are of limited value in identifying areas of risk to patients or solutions to the problem of avoidable deaths. In this study, to identify areas of service failure amenable to improvement through strengthened clinical policies, procedures, and practices, the researchers undertake a thematic analysis of deaths in hospitals in England that were reported by healthcare staff to a mandatory patient-safety-related incident reporting system. Since 2004, staff in the UK National Health Service (the NHS comprises the publicly funded healthcare systems in England, Scotland, Wales, and Northern Ireland) have been encouraged to report any unintended or unexpected incident in which they believe a patient's safety was compromised. Since June 2010, it has been mandatory for staff in England and Wales to report deaths due to patient-safety-related incidents. A thematic analysis examines patterns (“themes”) within nonnumerical (qualitative) data.

**What Did the Researchers Do and Find?:**

By searching the NHS database of patient-safety-related incidents, the researchers identified 2010 incidents that occurred between 1 June 2010 and 31 October 2012 that resulted in the death of adult patients in acute hospital settings. By scrutinizing the structured information in each incident report and the associated free text in which the reporter described what happened and why they think it happened, the researchers classified the reports into 18 incident categories. These categories fell into six broad areas of systemic failure—mismanagement of deterioration (35% of incidents), failure of prevention (26%), deficient checking and oversight (11%), dysfunctional patient flow (10%), equipment-related errors (6%), and other (12%, incidents where the problem underlying death was unclear). Management of deterioration, for example, included the incident categories “failure to act on or recognize deterioration” (23% of reported incidents), “failure to give ordered treatment/support in a timely manner,” and “failure to observe.” Failure of prevention included the incident categories “falls” (10% of reported incidents), “healthcare-associated infections” (also 10% of reported incidents), “pressure sores,” “suicides,” and “deep vein thrombosis/pulmonary embolism.”

**What Do These Findings Mean?:**

Although the accuracy of these findings may be limited by data quality and by other aspects of the study design, they reveal patterns of patient-safety-related deaths in hospitals in England and highlight areas of healthcare that can be targeted for improvement. The finding that the mismanagement of deterioration of acutely ill patients is involved in a third of patient-safety-related deaths identifies an area of particular concern in the NHS and, potentially, in other healthcare systems. One way to reduce deaths associated with the mismanagement of deterioration, suggest the researchers, might be to introduce a standardized early warning score to ensure uniform identification of this population of patients. The researchers also suggest that more effort should be put into designing programs to prevent falls and other incidents and into ensuring that these programs are effectively implemented. More generally, the classification system developed here has the potential to help hospital boards and clinicians identify areas of patient care that require greater scrutiny and intervention and thereby save the lives of many hospital patients.

**Additional Information:**

Please access these websites via the online version of this summary at http://dx.doi.org/10.1371/journal.pmed.1001667.

- The NHS provides information about patient safety, including a definition of a patient safety incident and information on reporting patient safety incidents
- The NHS Choices website includes several “Behind the Headlines” articles that discuss patient safety in hospitals, including an article that discusses the 2012 report of high hospital death rates in England, “Fit for the Future?” and another that discusses the Keogh review of the quality of care in the hospitals with highest death rates
- The US Agency for Healthcare Research and Quality provides information on patient safety in the US
- Wikipedia has pages on thematic analysis and on patient safety (note that Wikipedia is a free online encyclopedia that anyone can edit; available in several languages)

## Introduction

Inpatient deaths are an inevitable outcome for some acutely and chronically ill patients admitted to hospital. Analyses to uncover potentially avoidable deaths should be a necessary part of the surveillance of quality and safety in any healthcare system. Approaches to assessing unsafe care leading to death can be categorised into mortality and morbidity reviews [1], case-note reviews [2],[3], and system-wide outlier analysis [4]. In England, such analyses led to the following: the recognition of major failures in the standards of care in the children's heart surgery service at the Bristol Royal Infirmary [5], the identification of disturbing levels of poor care and neglect of elderly patients at Stafford Hospital (Mid Staffordshire NHS Foundation Trust) [6], and an urgent review of quality of care in 14 hospitals with high mortality rates [7]. Metrics such as the hospital standardised mortality ratio and the Summary Hospital-Level Mortality Indicator are now used to identify hospitals with potential patient safety problems [4]. However, what these measures cannot easily reveal are the service shortfalls that may trigger harm.

Presenting comparative data to healthcare organisations in the form of indices of mortality is of limited value in establishing avoidable factors in the cause of deaths. If a hospital is not an outlier for deaths, its management is likely to take the view that nothing further needs to be done. Alternatively, if it does have higher than expected mortality, initial investigation will focus on the validity of the data to try to show that the adverse finding is artefactual rather than real. The accompanying media pressure and public interest mean that the accountability stakes for chief executive officers and health boards are very high, and likely to be higher if legal criminal sanctions are implemented in the manner recommended by the Mid Staffordshire NHS Foundation Trust Public Inquiry [6]. When a hospital is presented statistically as an outlier for deaths, invariably a more clinically orientated review will then be undertaken to explore causes of death and to make judgements on whether the deaths are indeed “excessive” or “avoidable”.

What is missing from these contentious and conflicting viewpoints is a regular process of looking at the patterns and trends in deaths to yield actionable findings at the hospital and national level. Our study examines the deaths in acute hospital settings that are reported by healthcare staff as patient-safety-related, rather than routine hospital episode statistics. Relatively large numbers of these deaths are regularly reported as incidents, but such data have hardly been mentioned or examined in the public debates, even though the term “patient safety” has been repeatedly used by the media and health officials when referring to hospital mortality levels.

We reviewed all patient safety incident reports of deaths starting when their reporting became mandatory. The aim of the study was to classify the reported deaths to identify broad areas of service failure capable of being addressed by strengthening clinical policies, procedures, and practices.

## Methods

This study was part of a research programme at Imperial College London funded by the National Health Service (NHS) in England to develop incident reporting in the NHS. Ethical approval is in place for this programme from the Health Research Authority.

The study population was all adult deaths reported by NHS staff as patient safety incidents from hospitals in England over the period 1 June 2010 to 31 October 2012, 17 mo in total. 1 June 2010 was chosen as the start date because this was the date reporting of deaths due to patient safety incidents in the NHS in England and Wales became mandatory [8]. Prior to that, all patient safety incidents, whether causing death or otherwise, were reported on a voluntary basis (this remains the case for incidents involving no, low, or moderate harm). Between the quarter in which death reporting became mandatory (1 April 2010–30 June 2010) and the corresponding quarter 2 y later (1 Apr 2012–30 June 2012), overall incident reports increased by 17% (from 278,279 to 325,714) and reported deaths by 30% (from 698 to 912) [9].

Since 2004, staff from all parts of the NHS have been encouraged to make an incident report of any situation in which they believe that a patient's safety was compromised. A “patient safety incident” is defined as “any unintended or unexpected incident which could have, or did, lead to harm for one or more patients receiving NHS care” [10]. Most reports are first made to a local NHS organisation and then sent in batch returns by the organisation's risk manager to the national level. A small number are made by staff directly to the National Reporting and Learning System (NRLS). The information required in each incident report covers the following: demographic and administrative data; the circumstances of occurrence; a categorisation of causation; an assessment of the degree of harm as “no”, “low”, “moderate”, “severe”, or “death”; and action taken or planned to investigate or prevent a recurrence. These data are captured in a structured reporting form, but there is also a section of free text where the reporter is asked to describe what happened and why they think it happened. Data are anonymised to remove the names of patients and staff members. The detailed structure of the database has been described elsewhere [11].

The national database of patient safety incidents now contains around 10 million reports. For most of its existence, the NRLS was managed by an independent agency within the NHS called the National Patient Safety Agency. The agency was abolished in mid-2010 by the UK government as part of a cost-cutting policy to reduce the number of bodies operating at arm's length from the government [12],[13]. However, the NRLS has continued to function as before, managed by the same team of staff in a temporary home until the government decides on its long-term location.

The database was searched for patient safety incidents resulting in the reported death of an adult over the period of the study. The study population comprised a total of 2,010 such incidents involving patients aged 16 y and over in acute hospital settings. Incidents of deaths in obstetric and mental health units were excluded since staff are encouraged to report these to relevant standing confidential enquiries [14],[15].

We used thematic analysis for the qualitative aspects of this work [16]. Each incident report was reviewed by two of the authors (L. J. D. and S. S. P.) independently, and, by scrutinising the structured information together with the free text, a main reason for the harm was selected and recorded as one of 18 incident types: failure to act on or recognise deterioration; failure to give ordered treatment or support in a timely way; failure to observe; inpatient falls; healthcare-associated infections; pressure sores or decubitus ulcers; suicides; venous thromboembolism (VTE) or pulmonary embolus; medication error; misinterpretation or mishandling of test results; unexpected per-operative death; inappropriate discharge; poor or inadequate handover; unavailability of intensive treatment unit beds; necessary equipment failed or faulty; necessary equipment misused or misread by practitioner; necessary equipment not available; and other. The incident type categories were created based on an inductive approach [17] from the data, and the final terminology used for the categories was agreed upon by the authors based on prior knowledge of unsafe practices in the NHS. Each category reflected the most prominent contributory factors resulting in the occurrence of the incident. The assumption was that an approach of one-to-one mapping would be possible. Saturation was achieved after a review of 500 incidents. The final coding framework was then applied to all the incidents. A third reviewer was selected as an arbitrator where discrepancies arose between the classification of incidents by the two coders (L. J. D. and S. S. P.). Agreement between the two coders in classifying the incidents was good (Kappa = 0.87). Root cause analyses were not available.

After this thematic analysis, all incident reports were indexed and mapped onto the 18 categories and further collapsed into six areas of systemic failure. The incident types were grouped into this second level as shown in Table 1.

**Table 1:** Patient-safety-related adult deaths in NHS acute hospital settings: analysis of areas of systemic service failure.

Area of Service Failure	Incident Type	Number of Incidents	Percent of Incidents
**Mismanagement of deterioration**	Failure to act on or recognise deterioration	462	23%
	Failure to give ordered treatment/support in a timely way	130	6%
	Failure to observe	113	6%
	**All mismanagement of deterioration**	**705**	**35**%
**Failure of prevention**	Inpatient falls	206	10%
	Healthcare-associated infections	202	10%
	Pressure sores/decubitus ulcers	7	<1%
	Suicides	28	1%
	VTE/pulmonary embolus	87	4%
	**All failure of prevention**	**530**	**26**%
**Deficient checking and oversight**	Medication error	60	3%
	Misinterpretation or mishandling of test results	34	2%
	Unexpected per-operative death (immediately/within 24 hours)	124	6%
	**All deficient checking and oversight**	**218**	**11**%
**Dysfunctional patient flow**	Inappropriate discharge	78	4%
	Poor/inadequate handover	94	5%
	Unavailability of intensive treatment unit beds	25	1%
	**All dysfunctional patient flow**	**197**	**10**%
**Equipment-related errors**	Necessary equipment failed or faulty	16	<1%
	Necessary equipment misused or misread by practitioner	79	4%
	Necessary equipment not available	22	1%
	**All equipment-related errors**	**117**	**6**%
**Other**		**243**	**12%**
**Total**		**2,010**	**100**%

Covers a 17-mo period from 1 June 2010 to 31 October 2012, during which reports of deaths were mandatory.

Descriptive statistics were calculated, and examples of free text extracted. The free text extracts were edited to spell out abbreviations and acronyms in full, and to remove any potential identification details.

## Results

The types of incidents relating to mismanagement of deteriorating, acutely ill patients made up the single largest category of systemic service failure, accounting for 35% (705/2,010) of deaths (Table 1). The extract of free text from one of the incident reports in this area of service failure (Table 2) is typical of the other reports that staff, usually nurses, made. These incidents involved a mixture of factors that related to inappropriate clinical handling of acutely ill patients. Initial observations were often delayed or incomplete, and the intervals between subsequent observations were inappropriately long. Clinical review was often not timely, and/or decisions about potentially life-saving interventions were not made.

**Table 2:** Extracts of free text from patient safety incident reports of death.

Area of Service Failure	Extracts of Free Text from Patient Safety Incident Reports of Death
Mismanagement of deterioration	Presented to the emergency department at 1130 with abdominal pain, vomiting, confusion and a fall. Not triaged until 1215. At this point did not have a spot blood sugar check. Several hours later returned from having a chest X-ray that showed air under the diaphragm. Seen by surgeons. Observations appear not to have been repeated until 1950. Now has a blood sugar of 1.3 mmol/l and a blood pressure of 59/19 mm Hg. Referred to the intensive treatment unit. Intensive treatment unit concerned that the patient may have been shocked and hypoglycaemic for several hours before this was recognised.
Failure of prevention	Patient died from pulmonary embolus in hospital on day 12 of admission. On admission, VTE risk score was not performed which would have indicated that Fragmin [low molecular weight heparin] at a prophylactic dose should have been prescribed.
Deficient checking and oversight	Patient had chest X-ray and presented again, a year later, with a bronchial carcinoma and widespread disease and died five days later. Lung cancer judged to be present in the original chest X-ray and this was not included within the radiology report. The patient received no follow-up.
Dysfunctional patient flow	Sustained head and wrist injury whilst at garden centre. No loss of consciousness but had vomited once. Currently on warfarin. Glasgow Coma Scale = 15/15 on arrival in the emergency department. Seen by locum doctor; X-ray of the wrist did not reveal any fractures. No anticoagulation checks or request made for a CT scan. Discharged with minor head injury advice. Patient returned a day later with reduced consciousness and a CT scan revealed an intracranial bleed.
Equipment-related errors	Elderly patient with rheumatic heart disease who had a redo mitral valve repair. Decision made to replace Ryles nasogastric tube (for gastric drainage) with fine bore nasogastric tube. Chest X-ray reviewed by intensive treatment unit registrar and nasogastric tube deemed to be in correct position. Feeding commenced. Patient went into respiratory deterioration, was reintubated and ventilated. Nasogastric tube found to be in trachea and feeding stopped.
Other	Relatives informed that a body due for release for burial (Body A) was in fact that of another individual (Body B). Both bodies had been kept frozen for a long period of time and confusion over the correct body to be released had occurred.

As Table 1 also shows, amongst the incidents classified as failures of prevention (26%, 530/2,010), falls and healthcare-associated infections were the most frequent incident types, between them making up one in five of the reported deaths. The extract of free text from a sample incident (Table 2) demonstrates that the reporter was concerned that a national policy to prevent harm—in this case, VTE—had not been implemented, which resulted in the death of a patient.

Deficiencies in checking and oversight accounted for 218/2,010 (11%) of all incidents resulting in death. This heterogeneous group of systemic failures includes errors associated with unexpected per-operative death (6%, 124/2,010), medication errors (3%, 60/2,010), and misinterpretation or mishandling of test results (2%, 34/2,010) (Table 1). The most frequently occurring incident type in this area of service failure was unexpected per-operative death, and descriptions of events of this type included patients dying unexpectedly on the operating table or from complications that resulted in a re-operation within 24 h. Medication errors occurred at various points—administering, prescribing, and dispensing. Misinterpretation or mishandling of test results encompassed those incidents involving misinterpretation of findings, failure to take action on serious abnormalities, and neglect of follow-through (of the kind illustrated by the free text incident extract provided in Table 2).

Ten percent (197/2,010) of the incidents (Table 1) were judged to relate to dysfunctions in the way that patients flowed into, within, and out of the hospital. Temporary or sustained shortages of intensive care capacity were associated with one in 100 deaths reported, whilst 9% (172/2010) of incidents related to decisions and exchange of information on transfer between hospital departments, or from the hospital to the community. The sample incident extracted in Table 2 has features similar to others in that the reporter clearly attributes an inappropriate clinical decision to discharge a patient to clinical inexperience.

Failures in medical equipment, its misuse by practitioners, or its absence when urgently needed were associated with 6% (117/2,010) of deaths reported as patient safety incidents. The extract from an incident report in this area of systemic service failure (Table 2) shows how death resulted from a failure to use a standard piece of equipment according to the required procedure.

Overall, 12% (243/2,010) of incidents could not be classified. The category “other” contained a mixture of reports, some where the details were highly specific but did not fit any of the incident types (such as the serious mortuary mix-up described in the incident report extract in Table 2), and many others where the report was too vague or incomplete to deduce what the reporter was trying to convey.

## Discussion

How to achieve clarity on the utility of databases of patient safety incident reports remains an open debate [18]–[20]. One view holds that reporting systems are of value only in detecting rare events because extensive under-reporting prevents broader conclusions being made about the degree of harm to patients. An alternative view asserts that data from incident reports, despite their limitations, are the only data source through which to characterise harm so as to highlight areas of action to improve patient safety. Both views are important in that the true rate of harm cannot be established from reporting systems alone, yet information on types of harm is invaluable for identifying key areas of risk amenable to intervention. At the reporting level, it is also unclear to some reporters what sort of incidents should be reported and the level of detail required so that useful reporting and learning can occur locally and nationally. Whilst a formal definition is available to guide reporting to the database used in our study [8], it is very broad. This certainly keeps the threshold for deciding whether to report low, but perhaps does not encourage reflection and focus on precision when formulating the content of a report. Our analysis takes account of the limitations of incident reports but shows how, despite these, value can be extracted to yield clear insights into important sources of unsafe care at the system level.

Our finding that mismanagement of deterioration of acutely ill patients was a feature in more than a third of the patient-safety-related reported deaths points to an area of concern needing attention in the NHS. The underlying factors identified in previous NHS audits of acutely ill patients—weak organisation, inadequate knowledge, lack of clinical urgency, poor supervision, and failure to seek advice—echo the features of many of the incident reports that we reviewed [21],[22]. The National Patient Safety Agency highlighted the need to focus on deteriorating patients in 2007 [23]. The National Institute for Health and Care Excellence advocated several changes to this area of care: better recording of physiological variables, early warning scores, senior clinical input, and better handover [24]. In 2012, the Royal College of Physicians promoted its National Early Warning Score as a replacement for the multiplicity of such scores currently being used in the NHS [25]. Different scoring systems cause ambiguity and potential confusion amongst clinical decision-makers, particularly when staff move from hospital to hospital. A standardised early warning score, if mandated by the NHS, could save lives by ensuring more uniform identification of acutely ill patients. In addition, more timely responses (e.g., in the form of rapid response teams) will be needed to rescue acutely deteriorating patients.

Despite clear policy statements (Patient Safety First [http://www.patientsafetyfirst.nhs.uk/], 1000 Lives Plus [http://www.1000livesplus.wales.nhs.uk/], [26]) to encourage preventive programmes in well-established areas of harm—healthcare-associated infection, falls, VTE, inpatient suicides outside mental health services, and pressure ulcers—our data show that almost a quarter of all adult deaths reported as patient safety related are still associated with these causes. Progress has been made in some areas. Between 2006 and 2011, the prevalence of healthcare-associated infections in the NHS was reduced from 8.2% to 6.4%, and the prevalence of two key infections, methicillin-resistant *Staphylococcus aureus* and *Clostridium difficile* declined by 70% [27]. Since 2010, NHS hospitals have been required to implement measures to prevent VTE [28]. The quality of implementation across the whole service has not been established, but exemplar centres have shown that screening 95% of all patients is feasible [29]. The number of VTE-related deaths during the 17-mo period of our study—87—falls well below the widely promulgated estimate of deaths from this cause that triggered such concern that preventing VTE was made a national priority [30]. Potential explanations for this lower number of actual deaths could be the success of the VTE prevention programme in the NHS, or that the baseline assumption for VTE incidence was erroneously high, an observation that would be consistent with extrapolation of US epidemiological data to the UK population [31].

In contrast to the national priority given to reducing infection, VTE, and pressure sores, national priority has not been given in the same way to falls, a seemingly intractable cause of harm. Almost 208,000 inpatient falls are reported to the NHS every year [32]. We have shown that 10% of all adult deaths reported as being patient safety related are associated with falls. Yet, prevention of falls remains an elusive target for most hospitals, despite their associated risk and cost. High-risk patients need to be identified and interventions targeted accordingly—the use of ward-specific fall run-charts, reducing environmental hazards, providing personal alarms, and communicating patient risk across multidisciplinary teams can help to ameliorate this problem. Better staffing levels could also help [33].

Almost 11% of deaths in our study were due to deficient checking and oversight: unexpected per-operative death, misinterpretation or mishandling of test results, or a medication error. Avoidable factors and potential interventions in unexpected per-operative death and medication error have been extensively studied [34],[35], although they remain a regular feature of incident reports. Significant work has been undertaken in the area of medication errors, with interventions that have focused on frequent checks, pharmacist review, technologies, and information systems. These all have been introduced and evaluated, with mixed results. The whole field of misdiagnosis has remained under-researched until recently, and work is badly needed to establish the scale and scope of harm arising from this source, and the claims made for decision support technology as a way to reduce it [36]. However, we accept that our estimates from our classification might be conservative because failure to diagnose permeates various incident types.

Our finding of deaths associated with poor or inadequate handover and inappropriate discharge is not novel, and, indeed, transfers across boundaries are well-documented sources of risk in other sectors [37] as well as health [38]. For example, the erroneous discharge of patients from emergency departments back to the community is an ongoing problem that highlights unresolved dilemmas in clinical training as well as the level and seniority of staffing in these departments [39]. The precise reasons why erroneous discharge occurs require detailed local investigation. However, a more risk-averse approach to discharge would have resource implications arising from a greater number of precautionary admissions.

The NHS needs to pay greater attention to the equipment it procures, and its subsequent maintenance and proper use. We consider 117 (6%) deaths attributable to equipment-related failures to represent an important area the NHS can correct. The idea that patients should still be dying in the NHS from misplaced nasogastric tubes (our sample incident report in Table 2) despite several rounds of national guidance [40],[41] should itself be a source of concern for a service setting its sights on zero harm. The occurrence of faulty or failed equipment, a feature of some of the reports of death in our study—as well as our earlier work that showed that the number of device-related errors was rising compared with user-related errors in laparoscopic cholecystectomy [42]—must surely be one of the easier targets for risk elimination.

The inquiry into poor standards of care at Stafford Hospital [6] produced 290 recommendations in a searching assessment of the functioning of the entire NHS system in protecting patients and assuring the quality and safety of their care. Recommendations include the need to “develop and make more information available” from the NRLS, to “establish metrics” relevant to providing “strategic oversight” of patient safety, and to “enhance the use and analysis” of healthcare information. Our methodology makes substantial contributions in these areas. First, we have extracted more value than currently exists from the patient safety incident reports. Second, we have presented a framework of analysis that allows for strategic insight and surveillance to identify important systemic failures in the safety of care. Third, we address one of the underpinning themes of the Stafford Hospital inquiry: that greater visibility and transparency of information is essential to allow the public and patients to understand the risks of care.

### Strengths and Limitations

Any incident reporting system is limited by data quality, particularly the extent of under-reporting, selective reporting, and incomplete reporting. This has been a criticism of the NRLS in the past. However, our study population was deaths in a time period after reporting of incidents associated with death and severe harm became mandatory in the NHS. That is not to say that all such incidents will have been reported, but our study is likely to have captured a higher proportion than would have previously been the case. Moreover, the aim of our study was not to determine the absolute incidence of severe harm, but to identify situations where frontline staff perceived their patient's care to have been unsafe and associated with death. Although root cause analysis may have been available to supplement these incident reports, we lacked the resources to undertake a full review of these reports as we would have been swamped with very granular details that would likely have been difficult to aggregate. Alternatively, had we simply relied on the structured categories of causation in the reports, we would have been reliant on several hundred local reporters' and risk managers' opinions on the causes of the incidents.

Instead, our analysis takes account of those local perceptions, but is also based on detailed study of the free text (narrative) section of the incident reports. We acknowledge that the “true” cause of an incident is invariably multi-factorial, and we do not claim to have exposed the “true” cause. That would be impossible without detailed, and in many cases extremely extensive, investigation. However, we believe our approach is important in that it has identified major systemic features of clinical services that seem to be generating harm serious enough to cause death. It is a pragmatic rather than purist approach to using data conscientiously reported by concerned frontline staff that would otherwise lie fallow.

In the identification and characterisation of the incident types in the second level of our classification, there is at times the potential for some overlap between categories. For example, the incident type “pressure sores” has been placed into the “failure of prevention” area of systematic failure. It could be argued that if a hospital failed to document skin breakdown on admission (a factor that can result in pressure sores), the incident would better belong to the “deficient checking and oversight” area of systemic failure.

In our experience, incident reporting data cannot be used routinely to distinguish “avoidable” from “unavoidable” harm, although many reports, by the nature of the incidents, clearly fall into the former category. What is needed is recognition that when there is an accumulation of incidents in a particular area of care, this should trigger a response to ensure that patients are protected. The large number of incident reports and the urgency of many of the situations described means that the depth of investigation required to determine “avoidability” in each such case is just not feasible. For example, without standardised root cause analysis of each case, we cannot determine how many of the 2,010 deaths would have been avoided, but we can assume that the level of concern expressed by the staff making the reports has highlighted major problems. Moreover, the events described should not be happening in a modern, high-quality health service. Indeed, the staff reporting had a high enough level of concern to suggest that most of the deaths had an element of preventability.

### Conclusion

We have shown that analysing a national reporting system generating a very high volume of patient safety incident reports can point to recurring and actionable findings. The nature of the deaths occurring, associated as they are with common processes of care, should be a matter of great concern to a modern health service targeting “continuing reduction in harm” [43]. The positive feature of this finding is that there is an opportunity for corrective action to save many lives. The use of patient safety data in this way needs to become an everyday routine within the NHS at the national and local level, as well as have a role in shaping clinical decisions. Thus, there is an important role for specialty-specific teams or mortality review committees [44] to review their own incidents and implement solutions locally, and to draw attention to generalisable, national risk reduction action. Use of a classification system, such as the one proposed in this study, would allow hospital boards and clinicians to identify and prioritise areas for greater scrutiny and intervention. At present, there is a disconnect between national harm reduction initiatives, areas of concern that hospital staff see as important, and feedback between these levels. Focusing on actionable findings may be one way forward.

## Abbreviations

NHS: National Health Service
NRLS: National Reporting and Learning System
VTE: venous thromboembolism
